# The TOTEM RRMS (Testosterone Treatment on neuroprotection and Myelin Repair in Relapsing Remitting Multiple Sclerosis) trial: study protocol for a randomized, double-blind, placebo-controlled trial

**DOI:** 10.1186/s13063-020-04517-6

**Published:** 2020-06-29

**Authors:** Katline Metzger-Peter, Laurent Daniel Kremer, Gilles Edan, Paulo Loureiro De Sousa, Julien Lamy, Dominique Bagnard, Ayikoe-Guy Mensah-Nyagan, Thibault Tricard, Guillaume Mathey, Marc Debouverie, Eric Berger, Anne Kerbrat, Nicolas Meyer, Jérôme De Seze, Nicolas Collongues

**Affiliations:** 1 Centre d᾿Investigation Clinique INSERM 1434, Strasbourg, France; 2 Departement of Neurology, Hôpital de Hautepierre, University Hospital of Strasbourg, Strasbourg, France; 3 Departement of Neurology, Hôpital Pontchaillou, University Hospital of Rennes, Rennes, France; 4 Laboratory of Engineering Sciences, Computer Science and Imagery (ICube), CNRS, Institute of Biological Physics, University of Strasbourg, Strasbourg, France; 5 Departement of Myelin Biopathology, Neuroprotection and Therapeutic Strategies, UMR_S Inserm 1119, Strasbourg, France; 6 Departement of Urological Surgery, Nouvel Hôpital Civil, University Hospital of Strasbourg, Strasbourg, France; 7 Departement of Neurology, Hôpital Central, University Hospital of Nancy, Nancy, France; 8 Departement of Neurology, Hôpital Jean Minjoz, University Hospital of Besançon, Besançon, France; 9 Department of Neurology, Hôpital de Pontchaillou, University Hospital of Rennes, Rennes, France; 10 Departement of Public Health, GMRC University Hospital of Strasbourg, Strasbourg, France

**Keywords:** Multiple sclerosis, Testosterone, Neuroprotection, Remyelination, Randomized controlled trial

## Abstract

**Background:**

Central nervous system damage in multiple sclerosis (MS) is responsible for serious deficiencies. Current therapies are focused on the treatment of inflammation; however, there is an urgent need for innovative therapies promoting neuroregeneration, particularly myelin repair.

It is demonstrated that testosterone can act through neural androgen receptors and several clinical observations stimulated an interest in the potential protective effects of testosterone treatment for MS. Here, we sought to demonstrate the effects of a testosterone supplementation in testosterone-deficient men with relapsing-remitting MS.

**Methods/design:**

This report presents the rationale and methodology of TOTEM RRMS, a French, phase 2, multicenter, randomized, placebo-controlled, and double-blind trial, which aims to prevent the progression of MS in men with low testosterone levels by administration of testosterone undecanoate, who were kept under natalizumab (Tysabri®) to overcome the anti-inflammatory effect of testosterone. Forty patients will be randomized into two groups receiving either a testosterone treatment (Nebido®) or a matching placebo. The intervention period for each group will last 66 weeks (treatment will be injected at baseline, week 6, and then every 12 weeks). The main objective is to determine the neuroprotective and remyelinating effects of testosterone using tensor diffusion imaging techniques and thalamic atrophy analyses. As secondary objectives, impacts of the testosterone supplementation will be studied using other conventional and unconventional MRI parameters and with clinical outcomes.

**Discussion:**

The action of testosterone is observed in different experimental autoimmune encephalomyelitis models and epidemiological studies in humans. However, despite several preclinical data and some small clinical trials in MS, clear evidence for a therapeutic effect of hormone therapy is still missing. Therefore, our goal is to demonstrate the effects of testosterone therapies in MS. As there is no effective treatment currently available on fatigue in MS, careful attention should also be paid to secondary endpoints: fatigue, cognitive functions, and other symptoms that may improve life quality.

Assuming a positive outcome of the trial, this treatment could be considered as a new neuroprotective and remyelinating therapy in relapsing-remitting MS and could be applicable to other demyelinating diseases.

**Trial registration:**

ClinicalTrials.gov NCT03910738. Registered on 10 April 2019.

## Background

Multiple sclerosis (MS) is an autoimmune and demyelinating disease of the central nervous system (CNS) related to mechanisms of inflammation and neurodegeneration [1]. It is also known as the most common cause of neurological disability in young adults. The main clinical manifestations are related to inflammation and demyelination of the CNS [2] leading to profound alterations in the conduction of nervous messages. Currently, background treatments are exclusively based on controlling the inflammatory aspects of the disease using immunomodulators or immunosuppressants [3]. Thus, there is an urgent need to develop new innovative therapies that can help repair the damaged myelin. Indeed, it is shown that the absence of myelin repair is a major contributor to the outcome and evolution of MS to its progressive form [4].

It has been shown that estrogen and testosterone receptors are expressed in the lymphocytes and in the CNS resident cells, including neurons, astrocytes, and oligodendrocytes [5], which led to an interest in the role of sex hormones in MS as well as their use as treatment. An increased prevalence of certain autoimmune diseases in patients with hypogonadism has been suggested [6] and epidemiological and clinical studies have shown differences in the prevalence and progression of MS between men and women. Indeed, women are more affected than men with a sex ratio of about 3/1 for the recurrent form of MS [7–9]. But the pathology in men tends to be more severe and develop faster at a later age [10, 11] and is associated with a greater cognitive decline than women [12, 13]. Moreover, in women affected by MS, the occurrence of relapses decreases during the third trimester of pregnancy, whereas an upsurge in the 3 months following delivery related to the drop of hormonal secretions can be observed [14]. A recent publication described that lower testosterone levels are associated with higher disability in men with MS [15]. Such differences could be explained by a deleterious role of ovarian hormones in women, a protective role of testicular hormones in men, or deleterious or protective effects of sex chromosomes [16]. Therefore, these observations stimulated an interest for the potential protective effects of sex hormones in MS [17] and in particular for testosterone.

We hypothesized that administrations of testosterone for men with MS could prevent the progression of the disease because of its potential neuroprotective and promyelinating effects [18]. Patients remain treated with natalizumab (Tysabri®) during the trial to overcome the anti-inflammatory role of testosterone. Our primary objective is to evaluate the remyelinating and neuroprotective effects of testosterone over the course of 54 weeks on patients with RR-MS treated with natalizumab, combining thalamic atrophy analysis and tensor diffusion imaging techniques. Indeed, the gradual loss of brain volume is an important characteristic of MS. Physiologically, this rate of atrophy varies on average between 0.1 and 0.3% per year while it can reach 0.5 to 1% in patients with untreated MS [19, 20].

This study is justified by the original mechanism of action of testosterone acting on neuroprotection and remyelination in MS as well as its excellent tolerance. These modalities of action represent a new therapeutic path that has no equivalent in the current therapeutic arsenal used in MS. Assuming a positive outcome of the trial, this new treatment could be considered during the relapsing-remitting phase of the disease in men with low testosterone levels as a new neuroprotective and remyelinating therapy.

## Methods/design

### Study design

**Fig. 1:**
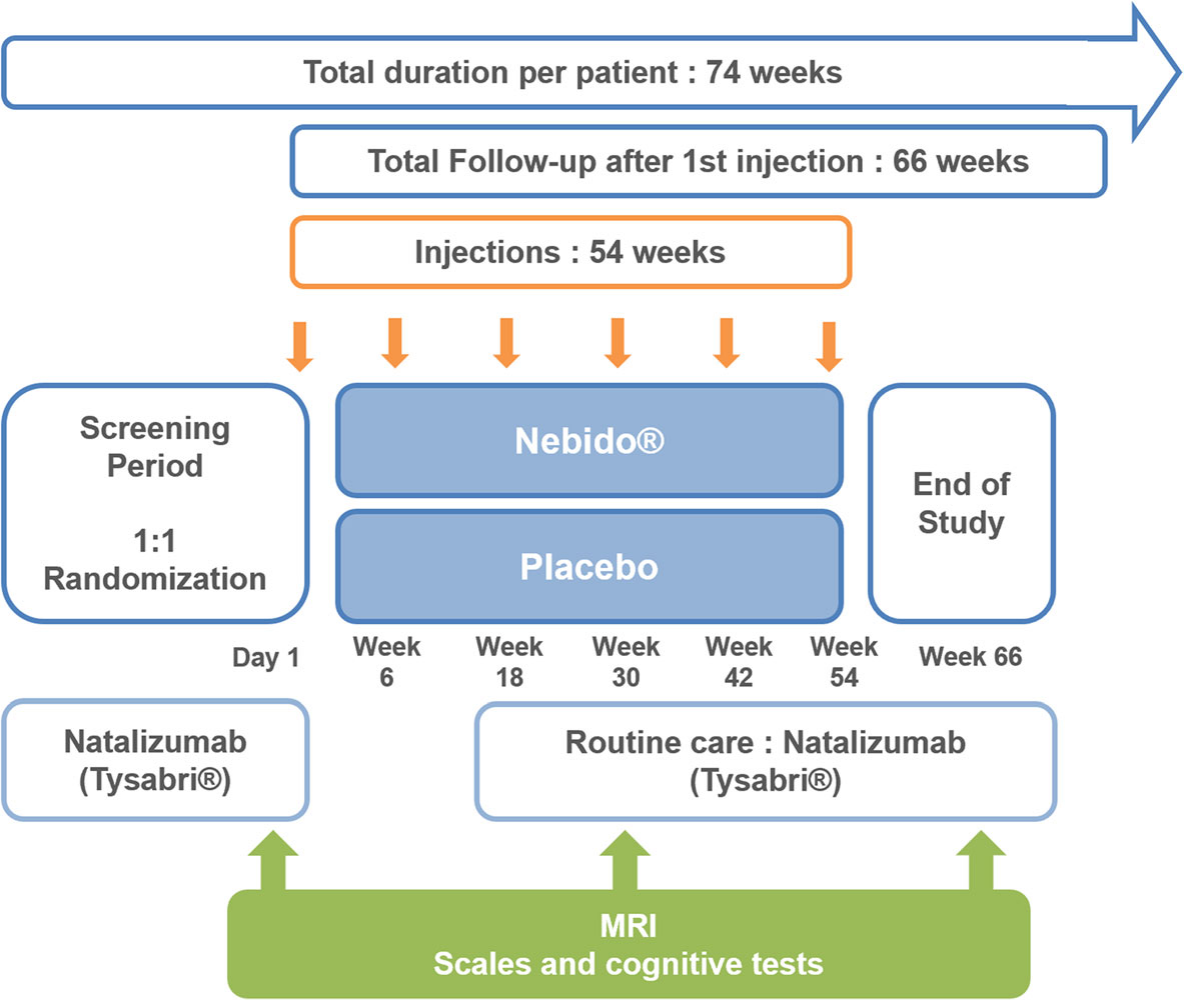
Flow chart of the trial process

The clinical study is a multicenter, randomized, 66-week, parallel-group, placebo-controlled phase 2 trial designed with double-blinded assessment and is being carried out in 40 testosterone-deficient men with relapsing-remitting MS. All participants will be randomly allocated to the intervention group or control group in a 1:1 ratio. The intervention group will receive the following treatment: “Nebido® testosterone undecanoate 1000 mg/4 ml solution for injection,” and the control group will receive the matching placebo treatment. Four milliliters of active treatment (Nebido® testosterone undecanoate) or placebo will be injected intramuscularly at baseline, at week 6, and then every 12 weeks (weeks 18, 30, 42, and 54). The total follow-up lasts 66 weeks after the first injection (Fig. 1).

The efficiency of the treatment will be evaluated by imaging measuring in both groups between inclusion and week 66 the evolution of conventional and unconventional MRI parameters. Clinical efficacy of the treatment will be evaluated by clinical outcomes including cognition, fatigue, quality of life, impact on work/activity, and anxiety/depression between inclusion and week 66. The safety of use and tolerance of the treatment will be evaluated by the number and nature of adverse events, evaluations of vital signs, clinical and neurological examinations, biological results, and locally interpreted MRI for tolerance and by monitoring concomitant medications (excluding Tysabri®).

### Inclusion and exclusion criteria for the trial

Patients who meet the following criteria will be deemed eligible for the trial: (1) man between 18 and 55 years; (2) affiliated to a social health insurance plan; (3) able to understand the objectives and risks related to the research and able to comply with the requirements of the protocol throughout the duration of the study; (4) informed of the results of the prior medical examination; (5) with a signed informed consent; (6) with confirmed and documented diagnosis of MS, as defined by the 2010 revised McDonald criteria; (7) treated with intravenous infusions of natalizumab (Tysabri®, 300 mg) once every 4 weeks for at least 1 year; (8) biological hypogonadism defined by serum testosterone levels below 15 nmol/L (checked by blood sampling during the inclusion visit); (9) negative status for JC virus or JC virus synthesis index ≤ 1.5 (confirmed by blood sampling at the inclusion visit, to ensure the maintenance of natalizumab during the study); (10) no relapses in the year prior to inclusion; (11) disability status during the selection visit with an EDSS score of 0 to 7 (verified by questionnaire during the inclusion visit); (12) stable neurological state in the month preceding randomization.

Patients who meet the following criteria will be excluded from the trial: (1) progressive MS (primary or secondary); (2) hypogonadism with clinical symptoms and treated with androgens; (3) PSA (prostate-specific antigen) > 2.5 ng/ml (for an age of less than 49 years) or > 3.5 ng/ml (for age ≥ 50 years) (confirmed by a blood test at the inclusion visit); (4) hemoglobin concentration > 16 g/dl (confirmed by blood sampling during the inclusion visit); (5) refusing or unable to undergo an MRI; (6) any other disease other than MS that may contribute to neurological symptoms and signs or affect their evaluation; (7) neurological signs compatible with progressive multifocal leukoencephalopathy (PML) or confirmed leukoencephalopathy; (8) untreated sleep apnea; (9) with or having had cancer or tumors of the liver, heart, kidney, prostate, or mammary gland; (10) cardiovascular, renal, hepatic, hematological, gastrointestinal, pulmonary, uncontrolled diseases; (11) wishing to procreate during the study period; (12) chronic infectious disease; (13) organic or psychiatric disease compromising their ability to understand the information given and to follow the protocol; (14) history of hypersensitivity to treatment or any of the excipients, or drugs of similar chemical classes; (15) used experimental drugs and/or participated in clinical drug trials in the 6 months prior to selection, (16) in exclusion period (determined by previous study or in progress); (17) impossibility of giving information to the patient (subject in emergency situation, difficulties in understanding the subject or other); (18) under tutors or curators; and (19) under the protection of justice.

### Recruitment

The participants will be recruited in France by neurologic units at the University Hospital of Strasbourg, Besançon, Nancy, and Rennes. After obtaining informed consent, participants are screened by a neurologist according to the inclusion and exclusion criteria. Those who satisfy all criteria are eligible to participate.

### Screening

As the main factor limiting inclusions being the serum testosterone level, the selection visit will be carried out in two stages. During the first visit at D-60, the testosterone level will be measured. If this level corresponds to the expected result under the protocol, the patient will be reviewed a second time to check the eligibility criteria. If the result of the total testosterone dosage does not correspond to the expected result, the patient will be withdrawn from the study and treated as part of the routine care.

Before the second visit at D-30, a PSA assay will be prescribed via a specific research prescription allowing the patient to carry out this analysis in a city laboratory. The major risk of taking Nebido® is the possible acceleration of pre-existing prostate cancer. A prostate examination will be carried out by the referring urologist of each center (secondarily to total testosterone and PSA dosages) for the detection of early prostate cancer or benign prostatic hyperplasia. Subjects who do not meet eligibility criteria cannot be randomized and will be withdrawn from the study. These patients will be followed as part of usual care.

### Randomization

The participants will be divided into the “treatment/Nebido®” arm or the “placebo” arm. Each eligible participant will be randomly assigned to either the intervention group or the control group according to the 1:1 equal proportion rule. The sequence of random allocation will be generated electronically via the Internet (Cleanweb®) by the investigator or one of the people in charge using personal access codes previously provided by the Data Manager.

### Sample size

The sample size is calculated using Bayesian methods. Subjects have about 30 to 35% chances of benefiting from an effect of the testosterone treatment with a halt in the development of their pathology. Placebo patients should not benefit from this effect. The sample size calculation is based on the following parameters:

- In the experimental group (Nebido®/testosterone), the expected proportion of subjects is modeled with a beta distribution (35, 65), indicating an expected proportion of success of 35%, with a standard deviation of 0.047 (or 4.7%).
- In the placebo group, the expected proportion of subjects is modeled with a beta distribution (5.95) indicating an expected proportion of success in the order of 5%, with a standard deviation of 0.022 (or 2.2%).

The success proportions of each group are then compared on the basis of the posterior distribution of the difference in proportions, using McMC methods, and the Nebido® is declared superior if the posterior probability that the difference between the two proportions is positive exceeds 0.95: Pr (*p*_T_ − *p*_P_ > 0) > 0.95.

The empirical type I error risk is 2%. With *N* = 20 subjects per group, over all simulations, the power of the test is of 80%. Thus, 40 subjects meeting eligibility criteria will be randomized, for a total of 80 subjects to be included in the study. The simulations were carried out with R 3.3.1.

### Blinding

The Bayer AG laboratory will be responsible for the blindness of treatment units. The experimental products (Nebido® and placebo) will be identical in appearance and conditioning to guarantee the blind. The indoor pharmacy of the University Hospital of Strasbourg will label the ampoules according to the certificate of conformity that has been provided for each batch by the Bayer laboratory. Transferring the treatments to the other centers will also be carried out by the pharmacy of the University Hospital of Strasbourg.

An independent statistician establishes the randomization list that is provided to the Data Manager and University Hospital of Strasbourg pharmacy. The pharmacy creates from this list a second list to establish a match between the study product defined by the randomization and the treatment number. This correspondence list gives no indication of the product delivered. The second list of numbered products is implemented in the e-CRF. At each randomization, the e-CRF delivers the number of the treatment to be administered to the patient.

In order to prevent unveiling, the results of the total testosterone assays will only be communicated and reviewed by the investigators after blind lifting.

### Baseline tests

The randomization visit takes place no later than 2 months after the selection visit for the patients who meet all the eligibility criteria. Before the injection of the experimental product, the following examinations are carried out:

- General clinical examination (vital signs, weight, height) and neurological examination, with medical history, concomitant treatment, and report of potential MS relapses
- Fasting blood test for lipid assessment and hormonal assay (free and total testosterone, TeBG, estradiol, cortisol, 17-OH progesterone, LH/FSH)
- EDSS score
- BICAMS, SF-36, EQ-5D, WPAI:MS, MFIS, and HADS questionnaires
- Conventional and unconventional brain MRI: DTI and/or NODDI

After the preparation of the experimental treatment (Nebido® or placebo), the administration is carried out by a slow intramuscular injection of the product (more than 2 min) in the gluteal muscle. The patient is kept under the supervision of a nurse for at least 1 h after the injection to monitor the possible occurrence of side effects related to the product. The same methods of injection and surveillance will be respected during all visits involving administration of these products.

### Study drugs

#### Study treatment

Testosterone undecanoate (Nebido®) is indicated as a replacement therapy for male hypogonadism when testosterone deficiency has been clinically and biologically confirmed. In this study, Nebido® will be used off-label in biologically testosterone-deficient patients but who do not necessarily have a clinical impairment as defined in the SPC. It is an intramuscular injectable solution of testosterone undecanoate at 1000 mg/4 ml in a clear yellowish oily solution. Nebido® is packaged in an amber glass ampoule containing 4 ml of solution corresponding to 631.5 mg of testosterone.

One thousand milligrams per 4 ml of study treatment or matching placebo (4 ml of oily solution containing the same excipients as Nebido®) will be injected intramuscularly over a 54-week period, according to the following schedule: at baseline, at week 6, and then every 12 weeks (at weeks 18, 30, 42, and 54). Patients are kept under the supervision of a nurse for at least 1 h after the injection of the experimental product to monitor the possible occurrence of side effects related to the product.

The latest data from the Bayer laboratory on the use of Nebido® and international publications show that it is not necessary to adjust the dose individually when the maintenance is carried out every 12 weeks [21–26]. This treatment regimen will allow excellent therapeutic compliance and to keep the testosterone levels blinded.

#### Auxiliary treatment

Natalizumab (Tysabri®) is used as an auxiliary treatment for MS background treatment and to overcome the anti-inflammatory role of testosterone, according to a use consistent with the SPC. Relapse treatment with Solumédrol® bolus (1 g × 3) or plasma exchange is authorized as well as the change of the immunosuppressive background therapy if necessary. Anticoagulant treatment or any other corticosteroid, immunosuppressive, or immunomodulatory therapy in addition to natalizumab (Tysabri®) will not be allowed during the trial, in order to study the testosterone’s specific effects.

### Follow-up

#### Clinical follow-up

All visits of the protocol, except for the first visit (week 6), are scheduled during the patient’s regular treatment visits (monthly injection of Tysabri®).

The follow-up is ensured by the patient’s neurologist and is scheduled at weeks 6, 18, 30, 42, 54, and 66. Adherence to the treatment and potential side effects will be monitored.

Assessment includes Nebido® testosterone undecanoate injection (weeks 6, 18, 30, 42, and 54); number and nature of adverse events; evaluation of vital signs; concomitant treatments; report of potential MS relapses; and EDSS scale, SF-36 questionnaire, EQ-5D-3L, WPAI:MS questionnaire, MFIS scale, and HADS questionnaire (baseline, week 30, and week 66) (Table 1).

**Table 1:** Study schedule

	Enrolment		Baseline	Treatment period					End of study
	Day - 60	Day - 30	0	Week 6	Week 18	Week 30	Week 42	Week 54	Week 66
**Informed consent**	X								
**Eligibility screen**	X	X	X						
**Tysabri® (routine care)**	X	X	X		X	X	X	X	X
**Clinical examination**	X		X	X	X	X	X	X	X
**Medical history**	X								
**Prostate examination by a urologist**		X							X
**Prostate-specific antigen**		X		X		X			X
Blood test (*fasting)	X	X	X^*^	X^*^	X^*^	X^*^	X^*^	X^*^	X^*^
**Hormonal dosage**	X		X		X	X	X	X	X
**MRI**			X			X			X
**Nebido®/Placebo**			X	X	X	X	X	X	X
**Scales and cognitive tests**			X			X			X
**AE/concomitant treatments**	X	X	X	X	X	X	X	X	X

#### MRI follow-up

Axonal and myelin repair is evaluated by using conventional MRI sequences (T1, T2 FLAIR) associated with a measure of diffuse and regional cerebral atrophy and by using advanced imaging methods (unconventional MRI sequences) such as diffusion tensor imaging (DTI), evolution of diffusion tensor imaging (NODDI), and quantitative magnetization transfer imaging (MPF). To determine the neuroprotective and remyelinating effects of the testosterone, MRI exams are scheduled at baseline, week 30, and week 66.

Conventional MRI sequences will evaluate the following: the T2 FLAIR lesion load according to an automatic segmentation based on a statistical model, evolution of this burden and lesions over time through a robust and proven MS imaging approach, volume and number of hypo-intense 3DT1 lesions, global atrophy evolution over time according to the SIENA method, and thalamus evolution over time according to the volumetry given by FreeSurfer.

Unconventional MRI sequences will evaluate the following: DTI images will be analyzed longitudinally using a multivariate tensor test; parametric maps provided by NODDI and MPF sequences will be analyzed longitudinally in order to quantify the zones showing a neuroprotective or remyelinating effect.

#### Biological follow-up

To monitor the potential effects of the testosterone treatment, patients will be subjected to blood and hormonal assays at each protocol follow-up visit. Blood samples will be anonymized before being sent to the local laboratory of each center or to city laboratories.

These analyses include NFS assay, liver function test (ASAT, ALAT, PAL, γGT, total and conjugated bilirubin), renal function test (urea, creatinine, glomerular filtration rate), fasting lipid test (total cholesterol, HDL, LDL, triglycerides), hormonal dosage (total and free testosterone, TeBG, estradiol, 17-OH progesterone, cortisol, LH/FSH) and total PSA assay (prostate-specific antigen) at baseline and weeks 1 to 6, and total testosterone dosage at the screening visit.

### Outcome measures

#### Primary outcome

The primary objective is to evaluate the remyelinating and neuroprotective effects of testosterone used for 54 weeks on patients with RR-MS, treated with natalizumab (Tysabri®), combining thalamic atrophy analyses and tensor diffusion imaging techniques.

The primary outcome is a binary criterion comparing the success rate in each treatment group, defined by course of thalamic atrophy lower than 0.5% and modification in transverse diffusivity of lesions lower than 0.5% per year, compared between baseline and week 66 in each group.

#### Secondary outcomes

The secondary objective is to study the impact of testosterone supplementation on other conventional and unconventional MRI parameters, on clinical outcomes (cognition, fatigue, quality of life, impact on work/activity, and anxiety/depression), and its tolerance by comparing results between the two groups and in each group between baseline, week 30, and week 66.

The secondary MRI endpoints are:

- Evolution of the number and volume of T1 hypo-intense lesions as detected by conventional MRI
- Evolution of the number and volume of new or enlarged T2 lesions as detected by conventional MRI
- Evolution of the total volume of hyper-intensity FLAIR lesion as detected by conventional MRI
- Evolution of diffusion tensor imaging (NODDI) as detected by unconventional MRI
- Evolution of quantitative magnetization transfer imaging (MPF) as detected by unconventional MRI

The secondary clinical endpoints are:

- Evolution of cognitive performance as measured by Brief International Cognitive Assessment for Multiple Sclerosis (BICAMS is a composite cognitive assessment tool comprising Symbol Digit Modalities Test (SDMT), California Verbal Learning Test-II (CVLT-II), and Brief visuospatial memory test—revised (BVMT-R))
- Changes in quality of life as measured by the SF-36 questionnaire
- Changes in quality of life related to health as measured by the European Quality of Life in 3 Dimensions (EQ-5D-3L) questionnaire
- Changes in work productivity and daily activities due to MS, as assessed by the WPAI:MS questionnaire
- Changes in fatigue, measured by the Multidimensional Fatigue Impact Scale (MFIS)
- Changes in anxiety and depression as measured by the Hospital assessment for Anxiety and Depression Scale (HADS) questionnaire
- Evolution of disability measured MS specific Expanded Disability Status scale (EDSS)
- Incidence of treatment-emergent adverse events (from visit 0/baseline to the end of the study (week 66))

### Data management

Patient data collection is the same in both arms. Data are collected at baseline, at week 6, and then every 12 weeks up to 66 weeks.

All data in this study will be rendered non-identifiable and then transcribed in an electronic observation book (e-CRF) by the investigator or a person designated by him. A record of any changes made to the notebooks will be kept. Consistency checks of the data collected will be made by computer according to predefined rules between the sponsor and the investigator described in the monitoring plan.

### Statistical analysis

#### Descriptive analysis

The statistical analysis will start with a description of the data. This descriptive analysis will be done in two successive ways. The description will first be made on the raw data and then as a second step from the Bayesian analysis of the parameters of interest. The description will be made on the a posteriori law of these parameters. The descriptive analysis will thus first provide a description of the sample data and second an estimation of the parameters of interest in the population.

The qualitative variables will be described by giving the frequencies and proportion of each modality. For the ordinal qualitative variables, the cumulated frequencies and proportions will be added.

The descriptive analysis will be univariate and bivariate. For the bivariate analysis, in addition to the frequencies, the proportions in rows, in columns, and in relation to the total of the contingency table considered will be given, crossing each variable of interest with the “treatment group” variable. Quantitative variables will be described using the usual positional parameters (mean, median, minimum, maximum, percentiles 1, 2.5, 5, 10, 25 (Q1), 75 (Q3), 90, 95, 97, 5, and 99) as well as the classical dispersion parameters (standard deviation, variance, range, interquartile gap). They will also benefit from a description first univariate then bivariate by level of the variable “group.”

#### Inference analysis

Statistical analysis of the primary endpoint will be done using a comparison of proportions, that is, the success rate in each treatment group. The success rates of each of the two groups will be modeled using beta distribution Be(*α*; *β*). The difference between the two proportions will be estimated on the posterior distribution of the difference between the two proportions (whose posterior probability density will be obtained by McMC algorithms). The difference between the two treatments will be concluded if the probability that the difference is positive exceeds 0.95: Pr (*p*_T_ − *p*_P_ > 0) > 0.95.

In this analysis, the prior distributions will be the following ones (with T = group “testosterone” and P = group “placebo”):

- *α*_T_ = 1; *β*_T_ = 1 and *α*_P_ = 1; *β*_P_ = 1: uniform distribution
- *α*_T_ = 0.5; *β*_T_ = 0.5 and *α*_P_ = 0.5; *β*_P_ = 0.5: Jeffreys’ prior distribution
- *α*_T_ = 3; *β*_T_ = 7 and *α*_P_ = 1; *β*_P_ = 9: optimistic prior distribution for the experimental treatment and pessimistic prior distribution for the reference treatment.

A logistic regression will then be adjusted to account for covariates and center effect (with a random effect in a mixed generalized linear model) or some characteristics of interest of the subject. The repeated data will be analyzed using mixed generalized linear models with the following effects: a fixed treatment effect, a fixed time effect, a random subject effect, and an interaction between the treatment and the time. As part of a sensitivity analysis, a random center effect will be introduced into the models. A Gaussian distribution will be used as a prior for the parameter of the group effect. Analysis of the secondary endpoints will be done according to the type of variable: when the judgment criterion is binary, the model is a logistic regression with random effects. Continuous outcome will be modeled with the linear model. The “score” type variables (bounded quantitative variables) will be analyzed with a beta regression model after a score transformation to the interval [0; 1] with a subject effect. In all these models, the “center” effect as well as the subject effect will be treated as a random. To model the time effect, two variants of the basic model will be adjusted: (1) a fixed slope and a random intercept model and (2) a random slope and intercept model.

#### Treatment of missing data and aberrant outliers

The missing data will be described. The number of missing data will be given for each variable individually and for all pairs of variables whose analysis is relevant in the context.

Multiple imputation will be used in each model with an imputation process depending on the type (MCAR, MAR MNAR) and frequency of missing data.

Outliers will be treated either by applying a mixture model with a specific law or by considering them as missing data if the analysis of their origin suspect a data entry error or an aberration in the measure.

## Discussion

The action of testosterone is being investigated in different EAE models and epidemiological studies in humans. However, despite several preclinical data and some small clinical trials in MS, a clear evidence of a therapeutic effect using hormone therapy is still missing. Along this line, hormone supplementation in MS patients cannot be currently recommended. Therefore, our goal is to demonstrate the therapeutic effects of a testosterone hormone therapy in MS through a phase 2 clinical trial.

Considering the potential side effects of these hormonal treatments and the risk of overdose, we decided that these treatments should be reserved for patients with hormonal deficits. It could also be argued that sex hormone synthesis is not limited to the gonads or adrenals but also occurs in the brain and therefore could be possible that sex hormone therapy might be beneficial even in individuals whose systemic hormone levels appear to be normal but who may have altered hormone synthesis and metabolism in the brain.

For testosterone, it has been shown that more than one third of male MS patients are testosterone deficient in serum and that the level of this deficit is correlated to their disability. No study has assessed estrogen levels in women with MS, but it is important to mention that the benefit in estrogen hormonal therapy has been mainly linked to estriol, which is only synthetized during the third trimester of pregnancy.

The difficulty that arises in the evaluation of these molecules as an add-on therapy is related to the confounding effect of an anti-inflammatory maintenance therapy. This confounding effect will not only be relevant for the anti-inflammatory effect but also for the remyelinating and/or neuroprotective effects that are also seen as secondary consequences of an anti-inflammatory treatment. For this reason, we chose to assess the neuroprotective and remyelinating effects of testosterone supplementation in patient without inflammatory activity in the brain, i.e., patients currently treated with natalizumab and who are good responders.

Therapeutic strategies targeting axonal and myelin repair face the challenge of assessing neuroprotection and remyelination in the CNS. To this end, we have chosen to evaluate these processes using conventional MRI sequences (T1, T2, FLAIR) associated with a measurement of diffuse and regional cerebral atrophy and so-called advanced imaging methods. Our primary endpoint is a combined one, which was chosen to obtain the most complete assessment of the neuroprotective and remyelinating components that are associated with the mode of action of testosterone. The literature shows that the assessment of thalamic atrophy seems to be the best marker of the neuroprotective effect in these patients [27–31]. This data can be extracted from standard sequences. Evolution of this atrophy in patients under natalizumab was evaluated around 1.5% after 48 weeks of treatment and then seemed to stabilize around 0.5% after this period [29, 32]. We therefore chose this as a threshold below which we defined a neuroprotective effect of testosterone. In addition, this rate is close to the natural course of brain atrophy which is 0.4% in healthy subjects.

We will use imaging methods for specific in vivo evaluation of myelin and axonal content including diffusion tensor imaging (DTI) and magnetization transfer imaging (MTI). DTI allows the characterization of tissue architecture and can be applied for the assessment of myelination. The radial diffusivity which represents the diffusion of water molecules perpendicular to the fiber bands corresponds to the integrity of the myelin [32, 33], whereas the axial diffusivity which is supposed to represent the diffusion of the molecules of water parallel to the fibers corresponds to the integrity of axons [34]. Given the small variation of transverse diffusivity in non-contrasting lesions after 1 year of natalizumab (0.11%/year on average), a decrease in its initial value of 0.5% will be considered significant to suggest remyelination [35]. DTI therefore has a particular interest in therapeutic trials to demonstrate the potential neuroprotective and remyelinating effects of the tested molecules. In this study, we plan to analyze the axial and radial diffusivity within T2 lesions. We should thus observe an increase in the axial diffusivity for the neuroprotective effect and a decrease in the radial diffusivity for the remyelinating effect [35–39].

Concerning MTI, this technique calculates an index, the ratio of magnetization transfer (MTR), that provides an estimate of the extent of the disturbance in tissue structures. Several studies have demonstrated a relationship between MTR and demyelination in the cerebral cortex as well as in the deep gray matter [40, 41]. Therefore, a low MTR reflects the reduced ability of macromolecules in the tissue to exchange magnetization with surrounding water molecules and is interpreted as an indication of damage to myelin and other cellular structures such as axonal membranes [41, 42]. In the same way, increases in the MTR are compatible with remyelination [36, 42, 43].

In addition, because there is no effective treatment currently available on fatigue in MS, careful attention should be paid to secondary endpoints for determining the effects of testosterone on fatigue, cognitive functions, and other symptoms that may contribute to the quality of life [18].

If the trial appears to be positive and that the neuroprotective and promyelinating effects of testosterone are confirmed, the treatment could be applicable to other demyelinating diseases as a new and innovative therapy.

## Trial status

The protocol version is number V1.2, and the date of this version is 23 March 2019. The trial started at the University Hospital of Strasbourg in November 2019. To date, 10 patients have been identified as testosterone deficient, and 4 of them support inclusion. The rate of enrolment will be around one patient per month. The other centers will be opened soon (University Hospital of Besançon, Nancy, and Rennes). Recruitment should end around September 2021.

## Data Availability

Hosting of the database coupled to the e-CRF CLEANWEB is managed by the company TELEMEDICINE Technologies S.A.S. having a set of dedicated virtual datacenters. This company ensures confidentiality, security, and data integrity, according to a security plan predefined by the Promoter and in accordance with international recommendations (ICH GCP part 11). IRM data will be sent to the CATI (authorization issued by the CNIL on 22/11/2016 no. 181590) by the participating centers in DICOM format that has been unidentified via a sftp server. The study data will be stored on a server hosted on the premises of the ICube laboratory (Institute of Biological Physics, Strasbourg, France). Their durability is ensured by a redundant storage on this server, as well as an off-site backup in the Ircad building (Strasbourg, France).

## Abbreviations

MS: Multiple sclerosis
RR-MS: Remitting-relapsing multiple sclerosis
EAE: Experimental autoimmune encephalomyelitis
MRI: Magnetic resonance imaging
CNS: Central nervous system
BICAMS: Brief International Cognitive Assessment for Multiple Sclerosis
SDMT: Symbol Digit Modalities Test
CVLT-II: California Verbal Learning Test-II
BVMT-R: Brief visuospatial memory test—revised
EQ-5D-3L: European Quality of Life in 3 Dimensions questionnaire
MFIS: Multidimensional Fatigue Impact Scale
HADS: Hospital assessment for Anxiety and Depression Scale
EDSS: Evolution of disability measured MS specific Expanded Disability Status
PSA: Prostate-specific antigen
PML: Progressive multifocal leukoencephalopathy
DTI: Diffusion tensor imaging
NODDI: Neurite orientation dispersion and density imaging
